# Lymphatic disorders caused by mosaic, activating *KRAS* variants respond to MEK inhibition

**DOI:** 10.1172/jci.insight.155888

**Published:** 2023-05-08

**Authors:** Sarah E. Sheppard, Michael E. March, Christoph Seiler, Leticia S. Matsuoka, Sophia E. Kim, Charlly Kao, Adam I. Rubin, Mark R. Battig, Nahla Khalek, Erica Schindewolf, Nora O’Connor, Erin Pinto, Jessica R.C. Priestley, Victoria R. Sanders, Rojeen Niazi, Arupa Ganguly, Cuiping Hou, Diana Slater, Ilona J. Frieden, Thy Huynh, Joseph T. Shieh, Ian D. Krantz, Jessenia C. Guerrero, Lea F. Surrey, David M. Biko, Pablo Laje, Leslie Castelo-Soccio, Taizo A. Nakano, Kristen Snyder, Christopher L. Smith, Dong Li, Yoav Dori, Hakon Hakonarson

**Affiliations:** 1 Center for Applied Genomics,; 2 Division of Human Genetics, and; 3 Zebrafish Core, Children’s Hospital of Philadelphia, Philadelphia, Pennsylvania, USA.; 4 Department of Dermatology, Perelman School of Medicine at the University of Pennsylvania, Hospital of the University of Pennsylvania, Philadelphia, Pennsylvania, USA.; 5 Richard D. Wood Jr. Center for Fetal Diagnosis and Treatment and; 6 Jill and Mark Fishman Center for Lymphatic Disorders, Children’s Hospital of Philadelphia, Philadelphia, Pennsylvania, USA.; 7 Genetic Diagnostic Laboratory, Department of Genetics, Perelman School of Medicine at the University of Pennsylvania, Philadelphia, Pennsylvania, USA.; 8 Department of Dermatology and; 9 Division of Medical Genetics, Department of Pediatrics, University of California, San Francisco, San Francisco, California, USA.; 10 Roberts Individualized Medical Genetics Center, Division of Human Genetics;; 11 Department of Pathology;; 12 Department of Radiology;; 13 Department of Surgery; and; 14 Dermatology Section, Children’s Hospital of Philadelphia, Philadelphia, Pennsylvania, USA.; 15 Center for Cancer and Blood Disorders, Children’s Hospital Colorado, Aurora, Colorado, USA.; 16 Division of Oncology, Cancer Center, Children’s Hospital of Philadelphia, Philadelphia, Pennsylvania, USA.

**Keywords:** Cardiology, Genetics, Cardiovascular disease, Molecular biology, Molecular genetics

## Abstract

Central conducting lymphatic anomaly (CCLA) due to congenital maldevelopment of the lymphatics can result in debilitating and life-threatening disease with limited treatment options. We identified 4 individuals with CCLA, lymphedema, and microcystic lymphatic malformation due to pathogenic, mosaic variants in *KRAS*. To determine the functional impact of these variants and identify a targeted therapy for these individuals, we used primary human dermal lymphatic endothelial cells (HDLECs) and zebrafish larvae to model the lymphatic dysplasia. Expression of the p.Gly12Asp and p.Gly13Asp variants in HDLECs in a 2‑dimensional (2D) model and 3D organoid model led to increased ERK phosphorylation, demonstrating these variants activate the RAS/MAPK pathway. Expression of activating *KRAS* variants in the venous and lymphatic endothelium in zebrafish resulted in lymphatic dysplasia and edema similar to the individuals in the study. Treatment with MEK inhibition significantly reduced the phenotypes in both the organoid and the zebrafish model systems. In conclusion, we present the molecular characterization of the observed lymphatic anomalies due to pathogenic, somatic, activating *KRAS* variants in humans. Our preclinical studies suggest that MEK inhibition should be studied in future clinical trials for CCLA due to activating *KRAS* pathogenic variants.

## Graphical abstract

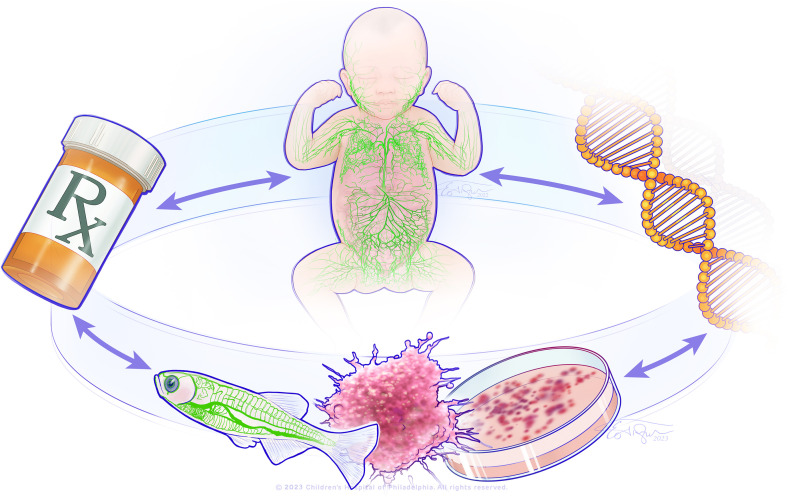

## Introduction

Lymphatic vessels function to resorb protein-rich fluid from tissues, transport immune cells, and return fluid and cells to the venous circulation (1). Congenital maldevelopment of the lymphatic system can result in a spectrum of clinical disorders with varying morbidity and mortality, including nonimmune fetal hydrops, chylous effusion (chylothorax), chylous ascites, and lymphedema. Complex lymphatic anomalies (CLAs) describe multiple disorders with diffuse lymphatic dysfunction and dilation, often with dynamic infiltration into organs and bone (2). Central conducting lymphatic anomaly (CCLA) is a rare CLA involving disrupted transit through the central lymphatics that can present with nonimmune fetal hydrops, chylothorax, chylous ascites, or lymphedema (3). Fewer than 25% of individuals with lymphedema and roughly 25% of individuals with CCLA have a genetic etiology identified to explain their maldeveloped lymphatics (4, 5).

In this study, we define the lymphatic phenotype and clinical course of 4 individuals with CCLA identified to express somatic pathogenic *KRAS* variants, including 3 with epidermal nevus syndromes. KRAS is an RAS family small guanine nucleotide binding protein (reviewed in ref.^6^). When bound by GTP, KRAS activates MAPK cell signaling to modulate cell growth and proliferation (reviewed in ref. 6). Mosaic, pathogenic *KRAS* variants have previously been reported to cause a range of epidermal nevus syndromes, including encephalocraniocutaneous lipomatous syndrome (ECCL) (MIM 613001), nevus sebaceous syndrome (NSS) (MIM 163200), and oculoectodermal syndrome (OES) (MIM 600268), as well as Gorham-Stout disease, a subtype of CLA (7–13).

For patients with CLAs, there is an urgent need to identify precision-based therapies that treat the underlying disorder. Standard treatment modalities for CLA and CCLA include surgical and interventional radiological procedures such as lymphovenous anastomosis or glue embolization. While often able to address acute symptoms, these procedures may not be curative and repeat interventions may be necessary to achieve adequate symptom control. Some patients may not be eligible for interventions. Moreover, these procedures do not address the underlying molecular difference. Use of targeted pharmacologic interventions, including inhibitors of the RAS/MAPK pathway and p110α catalytic subunit of phosphatidylinositol-3-kinase (PIK3CA)/AKT/mTOR pathway, have been reported, but few prospective clinical trials of these medications have been published (14–16). Sirolimus, an mTOR inhibitor, was the first targeted pharmacotherapy agent widely studied for symptomatic lymphatic anomalies (15–19). Thought to inhibit an inappropriately activated PIK3CA/AKT/mTOR pathway, sirolimus has been utilized for over a decade to reduce the functional impact of lymphatic anomalies. However, only a subset of patients respond to this treatment, and the impact is often incomplete. The efficacy of trametinib, a MEK1/2 inhibitor, has been demonstrated in a single patient case of RAS-related CLAs and in a *KRAS*-driven mouse model of Gorham-Stout disease (12, 20–23).

Given previous studies, we postulated the *KRAS* variants that are activating and repurposing targeted inhibitors previously used for cancer would allow us to test and precisely identify molecularly targeted novel therapeutics. We present our preclinical data to aid in the treatment of CLAs due to mosaic *KRAS* variants. Expression of activating *KRAS* variants in primary human dermal lymphatic endothelial cells (HDLECs) and zebrafish mimicked the lymphatic dysplasia seen in human study individuals and was rescued by MEK inhibition. These studies are a first step toward clinical trials evaluating the efficacy of MEK inhibitors for CLAs.

## Results

We identified 4 individuals with diffuse lymphatic disorders due to mosaic *KRAS* variants, including 3 with epidermal nevus syndromes (Table 1 and Supplemental Figure 1; supplemental material available online with this article; https://doi.org/10.1172/jci.insight.155888DS1).

### Individuals.

**Figure 1:**
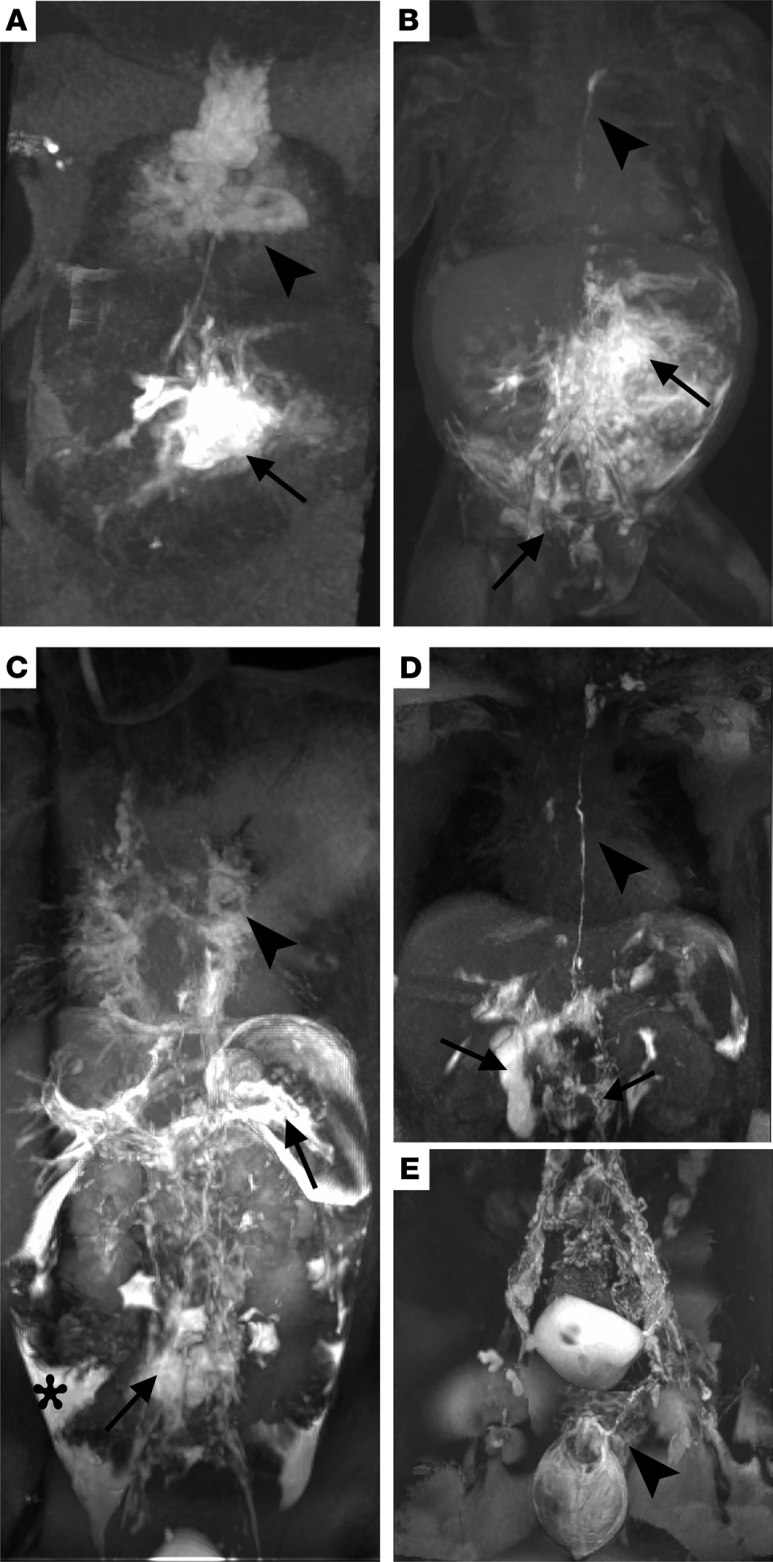
DCMRL of individuals 1, 2, and 4. (**A**) Intrahepatic DCMRL of individual 1 illustrating retrograde mesenteric perfusion (arrow) and pulmonary lymphatic perfusion with dilated mediastinal lymphatics (arrowhead). (**B**) Intranodal DCMRL of individual 1 shows retrograde into the mesentery (arrow), right renal, and dermal lymphatics (arrow), with an i.p. leak and intact thoracic duct coursing to the left venous angle (arrowhead). (**C**) Intrahepatic DCMRL of individual 2 shows retrograde flow into the lumbar and iliac lymphatics (arrow), splenic lymphatics (arrow), and i.p. leak (*). There is also bilateral pulmonary and mediastinal lymphatic perfusion (arrowhead) without a thoracic duct. (**D**) Intrahepatic and (**E**) intranodal DCMRL of individual 4 show a normal-appearing thoracic duct (arrowhead) with retrograde lumbar perfusion (arrow) and intraduodenal leak (arrow). Intranodal DCMRL shows dilated iliac lymphatics with retrograde dermal perfusion (scrotal and penile).

Individual 1 was an infant male of Puerto Rican and European ancestry who presented with a hybrid disorder consisting of both NSS and ECCL. He was previously reported as part of a larger cohort study (4). He was born at 33 weeks 4 days gestational age due to worsening nonimmune fetal hydrops following a pregnancy complicated by polyhydramnios, fetal arrhythmia, and multiple congenital anomalies. Postnatal evaluation demonstrated nevus psiloliparus on the scalp, biopsy-confirmed nevus sebaceous on the face and body, and an eyelid coloboma with a lateral epibulbar choristoma (Supplemental Figure 1, A, B, D, and I). Postnatal MRI showed microcystic right kidney, osseous thinning of the skull, choroidal osteoma, subpial spinal lipoma, and hemimegalencephaly (Supplemental Figure 1, G and H). Dynamic contrast MR lymphangiogram (DCMRL) at 3 months old demonstrated CCLA, including abnormal lymphatic leakage and contrast accumulation in the right renal hilum, retroperitoneum, mesentery, left pleural cavity, and back flow into both inguinal/lower abdomen s.c. soft tissues (Figure 1, A and B). There were abnormal transverse relaxation time hyperintense lesions within the mediastinum, as well as an abnormal signal extending from both hila into the periphery of the lungs and supraclavicular and neck soft issues that had the appearance of, and were suspected to be, abnormal lymphatic tissue or lymphatic malformations. Repeat lymphangiogram at 6 months of life showed new findings of retrograde lymphatic perfusion to the perihilar and pulmonary lymphatics bilaterally. A pathogenic variant in *KRAS* p.Gly12Asp (c.35G > A, NM_004985.3) at 23% variant allele frequency (VAF) was found in the nevus sebaceous and absent in leukocytes on the Nevus Gene Set. Ectopic multifocal atrial flutter was refractory to synchronized cardioversion. Subsequent control was achieved with propranolol, intermittent esmolol infusions, and digoxin. His hospital course was complicated by surgical necrotizing enterocolitis with ileostomy. Due to his tenuous clinical status, he was deemed to not be a candidate for medical management. He had worsening head and neck edema refractory to drain placement, thrombocytopenia, and peritonitis, and the decision was made to pursue comfort care. He died at 7 months old, prior to the completion of these studies.

Individual 2, a female child of European ancestry, presented with oculoectodermal syndrome including aplasia cutis congenita, epibulbar dermoids, and hyperpigmentation following lines of Blaschko. She was previously reported (9). A nonossifying fibroma (NOF) of the bone identified a mosaic pathogenic variant in *KRAS* p.Gly13Asp (c.38G > A, NM_033360.3) with 32.9% VAF by exome sequencing as was previously reported (9). The variant was identified in subsequent biopsies of hyperpigmented skin, periosteum, muscle, and humerus NOF samples (VAF ranging 10.3%–38.8%) and was not identified in blood and bone marrow (8). After her initial diagnosis, she saw a cancer geneticist who recommended annual complete blood counts until 5 years old and possible earlier screening for colon cancer. She also has middle cerebral artery stenosis and juvenile rheumatoid arthritis. At 9 years old, she developed chylous effusions and protein-losing enteropathy. At this time, sirolimus was the most common agent being used for patients with presumed lymphatic anomalies causing chylous effusions; thus, sirolimus was prescribed to maintain a trough level of 10–15 based on previous studies (15). DCMRL at 12 years old showed trace pleural effusions with extensive pulmonary interstitial fluid, trace pelvic ascites, and peritoneal spillage on initial injection consistent with a diagnosis of CCLA and pulmonary lymphangiectasia (Figure 1C). She previously was treated with ulixertinib (an ERK inhibitor), but this was stopped because of shortness of breath and worsening pulmonary disease.

Individual 3 is a male child of European ancestry with nevus sebaceous syndrome initially evaluated at 3 weeks of age for congenital chylothorax. The infant was evaluated at 3 weeks old for extensive right-sided epidermal nevus, cardiac arrhythmia, bilateral corneal opacities, and congenital chylothorax. He was born at 32 weeks gestational age via emergency Cesarean section due to fetal atrial tachycardia. He was unresponsive to cardioversion and his arrythmia was controlled on digoxin, flecainide, and propranolol. An initial echocardiogram was normal but he was subsequently found to have an atypical coarctation of the aorta. His chylothorax required chest tube drainage and improved with octreotide. No lymphatic imaging has been performed. Physical examination demonstrated extensive right-sided verrucous skin-colored plaque following lines of Blaschko with whorled appearance on the scalp, forehead, chest, suprapubic area, scrotum, and the upper and lower extremities, and membranous aplasia cutis congenita on the vertex scalp (Supplemental Figure 1, C, E, and F). Biopsies from 2 locations on the scalp showed nevus sebaceous and rudimentary meningocele. A somatic pathogenic variant in *KRAS* p.Gly12Val (c.35G > T, NM_004985.3) at 29% VAF was identified from nevus sebaceous but was absent in the blood on the gene panel. He subsequently developed intractable seizures requiring partial lobectomy. He also developed hemihypertrophy with bone mass on right humerus showing a benign moderately cellular spindle cell lesion containing scattered multinucleated giant cells. FGF23 FISH testing performed to evaluate for phosphaturic mesenchymal tumor was negative. He developed dependent edema of the right leg, thought to be lymphedema. A venous doppler performed at 2 years old only showed a dilated superficial calf vein. He is currently 6 years old and doing well.

Individual 4 is a 17-year-old young man of European ancestry with left lower extremity edema and chylous scrotal leak. He described trauma to the pelvic area with bruising and scrotal edema around 6 years old. Around 10 years old, his family noticed that his left calf was bigger than his right. MRI at that time showed increased signal in the s.c. fat and increased fluid in the soft tissues consistent with lymphedema. Around 12 years old, he developed a chylous scrotal leak. Prior to his presentation at our center, he tried compression to treat the lymphedema. This was discontinued as it worsened the scrotal leak. At 15 years old, he had an omental lymph node transfer and lympho-venous anastomosis to the superficial circumflex iliac vein to reroute flow from the left scrotum, which did not provide significant improvement. Topical sirolimus slightly improved his scrotal leak. He presented to our center for lymphatic imaging and intervention at 17 years old. His physical exam was notable for macrocephaly, large edematous-appearing scrotum with presence of microcysts, and an edematous left leg without pitting. DCMRL following intranodal injection showed reflux of contrast into prominent channels predominantly within the left inguinal region perfusing the scrotum, right and left hemipelvis (Figure 1, D and E). Additionally, there was a large 4 mm channel noted within the posterior thigh anterior to the semimembranous muscle visualized on initial imaging, likely due to retrograde flow from the intranodal injection. There was asymmetric edema involving the left lower extremity to about knee level with multiple prominent lymphatic channels visualized superficially. Interstitial lymphangiogram revealed filling of the channels predominantly anteriorly with progression of contrast to the level of the knee. For management, lymphovenous anastomosis from 4 lymphatic vessels in the thigh to the saphenous vein was performed and glue embolization of the left inguinal and bilateral scrotal lymphatic channels was performed. Biopsy of the scrotal area showed keratinized squamous epithelium with irregular, dilated lymphatic vasculature, highlighted by IHC stains D2-40 and CD31, and underlying smooth muscle consistent with a microcystic lymphatic malformation (Supplemental Figure 1, J–M). Somatic Overgrowth and Vascular Malformation Panel Version 3 and research exome sequencing identified a mosaic pathogenic variant in *KRAS* p.Ala146Thr (c.436G > T, NM_033360.4) at 3.3%–3.4% VAF in the scrotal tissue.

We hypothesized the *KRAS* variants were the etiology of the lymphatic anomalies for these individuals. To test this hypothesis, we used HDLECs and zebrafish to model the *KRAS* variants. To identify the most optimal medical therapy for individuals with mosaic *KRAS*-activating variants, we screened multiple therapeutics.

### In vitro modeling.

**Figure 2:**
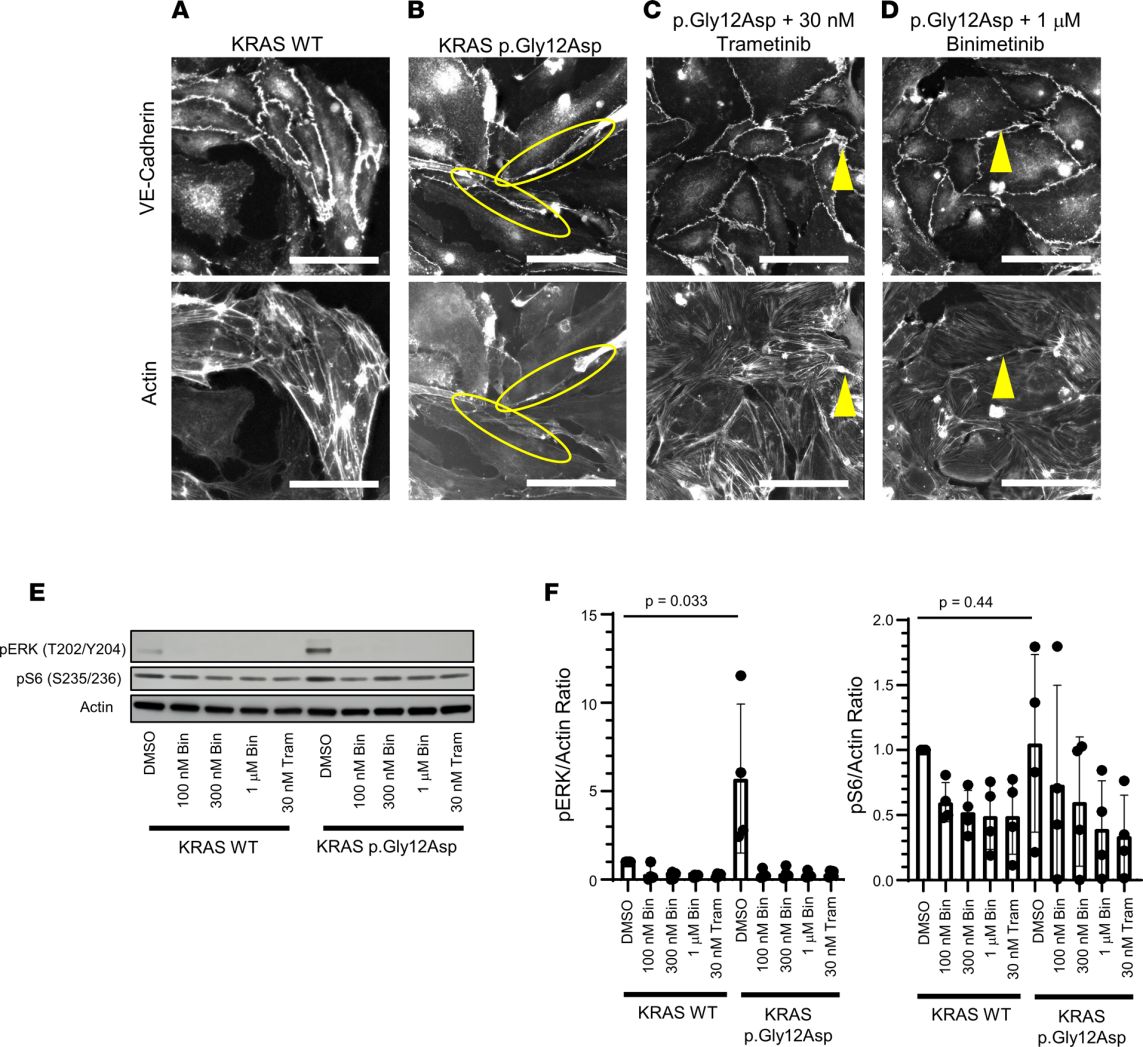
A 2D in vitro model of lymphatic dysplasia. HDLECs were stained with VE-cadherin or actin. Scale bars: 50 μm. (**A**) KRAS WT. (**B**) KRAS p.Gly12Asp. Yellow circles show extensions from cells. (**C**) KRAS p.Gly12Asp treated with 30 nm trametinib. (**D**) KRAS p.Gly12Asp treated with 1 μm binimetinib. Yellow arrows show areas where abnormal extensions remain. (**E**) Cell lysates from HDLECs transduced with either KRAS WT or p.Gly12Asp were analyzed with IB for pERK at T202 and Y204 or pS6 at S235/236 with actin as control. (**F**) Quantification of IB, pERK, or pS6 normalized to actin, normalized to WT + DMSO sample. Data were quantitated from 4 independent experiments. Bars are means; error bars are SDs. One-sided Student’s *t* tests were performed to calculate significance. Bin, binimetinib; Tram, trametinib.

Based on previous studies, we hypothesized the *KRAS* variants were activating and that an increase in MAPK signaling caused the lymphatic anomalies in our patients. HDLECs were transduced with human WT *KRAS*, *KRAS* p.Gly12Asp (Figure 2), or *KRAS* p.Gly13Asp (Supplemental Figure 3) and then stained with vascular endothelial cadherin (VE-cadherin) to evaluate endothelial cell junction integrity or actin to evaluate the cytoskeleton. Expression of *KRAS* variants following transductions was determined by quantitative PCR (qPCR) (Supplemental Figure 2A), and expression of the variants was found to be within a 2-fold range of the WT. Expression of *KRAS* WT had no impact on cell activation when compared with empty vector transductions in multiple readouts (Supplemental Figure 2, B–D). HDLECs expressing p.Gly12Asp had increased cellular extensions and protrusions, including growth over or under neighboring cells, and loss of normal organization of the actin cytoskeleton compared with HDLECs expressing WT *KRAS* (Figure 2, A and B) — a phenotype suggestive of the lymphatic dysplasia seen in individual 1 (Figure 1). Indeed, HDLECs expressing the p.Gly12Asp variant had a significant increase (~6×, *P* = 0.033) in phosphorylation of ERK compared with WT (Figure 2, E and F). There was a slight, nonsignificant (~1.1×, *P* = 0.44) increase in S6 phosphorylation, a marker of PI3K/AKT/mTOR signaling, in HDLECs expressing KRAS p.Gly12Asp compared with WT (Figure 2, E and F), suggesting the increase in MAPK signaling may contribute more to the lymphatic dysplasia than PI3K/AKT/mTOR signaling. The variability in ERK and S6 phosphorylation could be due to differences in transduction efficiency.

Given that transient transduction of *KRAS* p.Gly12Asp led to morphologic changes in HDLECs, including apparent loss of contact inhibition, we next sought to perform preclinical studies to identify molecularly targeted rescue therapy. We hypothesized that inhibition of MAPK signaling would rescue the abnormal cellular morphology. Trametinib (GSK1120212) is an oral non–ATP-competitive MEK1 and MEK2 inhibitor previously approved for combinatorial therapy in melanoma and efficacious in patients with CLAs (20–25) (Supplemental Table 1). Similar to trametinib, binimetinib is an oral non–ATP-competitive MEK1 and MEK2 inhibitor previously approved for combinatorial therapy in melanoma (26) (Supplemental Table 1). The morphological changes in HDLECs were reversed with treatment by trametinib or binimetinib (Figure 2, C and D). Treatment with trametinib or binimetinib decreased ERK phosphorylation (Figure 2, E and F) and S6 phosphorylation (Figure 2, E and F) to WT levels. Transduction of *KRAS* p.Gly13Asp led to similar biochemical and morphological changes, which were rescued by MEK inhibition (Supplemental Figure 3).

**Figure 3:**
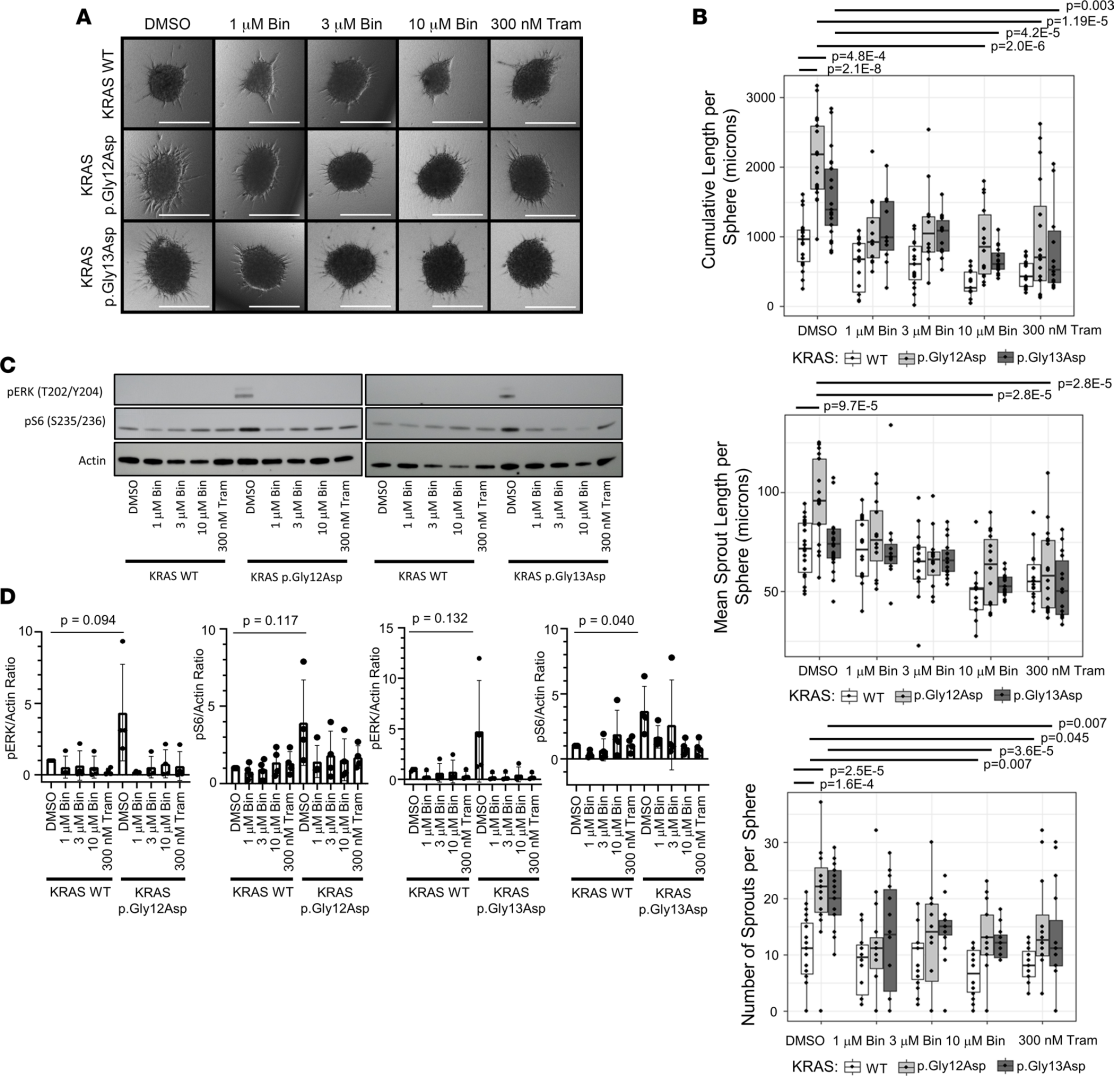
In vitro organoid model. (**A**) Lymphatic organoids were transduced with KRAS WT, KRAS p.Gly12Asp, or KRAS p.G13D and treated with DMSO (control), 1 μM binimetinib, 3 μM binimetinib, 10 μM binimetinib, or 300 nM trametinib. Scale bars: 300 μm. (**B**) Quantitation of sprouting data from 3 independent experiments showing cumulative sprout length per sphere (top), mean sprout length per sphere (middle), and number of sprouts per sphere (bottom). In the box and whisker plots, the center line is the median, the lower and upper boundaries of the box are the 25% and 75% quartiles, and the whiskers extend to 1.5 times the interquartile range from the 25% and 75% quartiles. Two-sided Student’s *t* tests were performed to calculate significance. Comparisons were made between DMSO-treated WT and both DMSO-treated mutants, as well as each DMSO-treated mutant and all the drug treatments of that mutant; *P* values were corrected for multiple testing with the Benjamini and Hochberg FDR method. Bin, binimetinib; Tram, trametinib. (**C**) IB from in vitro organoid model. Cell lysates from HDLECs transduced with either KRAS WT, p.Gly12Asp, or p.Gly13Asp were analyzed with IB for pERK at T202 and Y204 or pS6 at S235/236 with actin as control and quantified. (**D**) Quantification of IB from 4 separate experiments, pERK or pS6 normalized to actin, normalized to WT + DMSO sample. Bars are means; error bars are SDs. Bin, binimetinib; Tram, trametinib.

After verifying that transduction of *KRAS* p.Gly12Asp or *KRAS* p.Gly13Asp led to loss of normal endothelial cell junctions and disorganized actin cytoskeleton, we sought to understand the effects of the same variants on lymphangiogenesis. To do this, we used a 3D organoid model. Spheroids of HDLECs were created, embedded in collagen, and observed for sprout formation and elongation (Figure 3A). In this model, transduction of *KRAS* p.Gly12Asp or *KRAS* p.Gly13Asp led to a significant increase in cumulative sprout length per sphere compared with WT (*P* = 2.1 × 10^–8^; *P* = 4.8 × 10^–4^) (Figure 3B). Transduction of *KRAS* p.Gly12Asp but not *KRAS* p.Gly13Asp resulted in a significant increase in mean sprout length per sphere compared with WT (*P* = 9.7 × 10^–5^; *P* = 0.453), suggesting there may be some biological differences between the impact of these neighboring *KRAS* variants (Figure 3B). However, both *KRAS* p.Gly12Asp and p.Gly13Asp resulted in an increase in the total number of sprouts per sphere (*P* = 1.6 × 10^–4^; *P* = 2.5 × 10^–5^) (Figure 3B).

Next, we examined the effect of MEK inhibition on sprouting in the organoid model. MEK inhibition with 10 μm binimetinib or 300 nM trametinib significantly reduced cumulative sprout length in the *KRAS* p.Gly12Asp and p.Gly13Asp models, mean sprout length in the *KRAS* p.Gly12Asp model, and the number of sprouts per sphere in the *KRAS* p.Gly12Asp and p.G13D models, demonstrating near-complete rescue (Figure 3B). MEK inhibition with trametinib or binimetinib resulted in a reduction in phosphorylation of ERK and a reduction in S6 phosphorylation (Figure 3, C and D).

### In vivo modeling.

The zebrafish is an excellent in vivo system to study lymphatic development due to optical transparency and transgenic lines (27–29). Additionally, the large clutches of zebrafish are favorable for drug screening (30). Thus, we elected to use the zebrafish model to evaluate the effect of KRAS p.Gly12Asp and p.Gly13Asp on lymphatic development in vivo.

**Figure 4:**
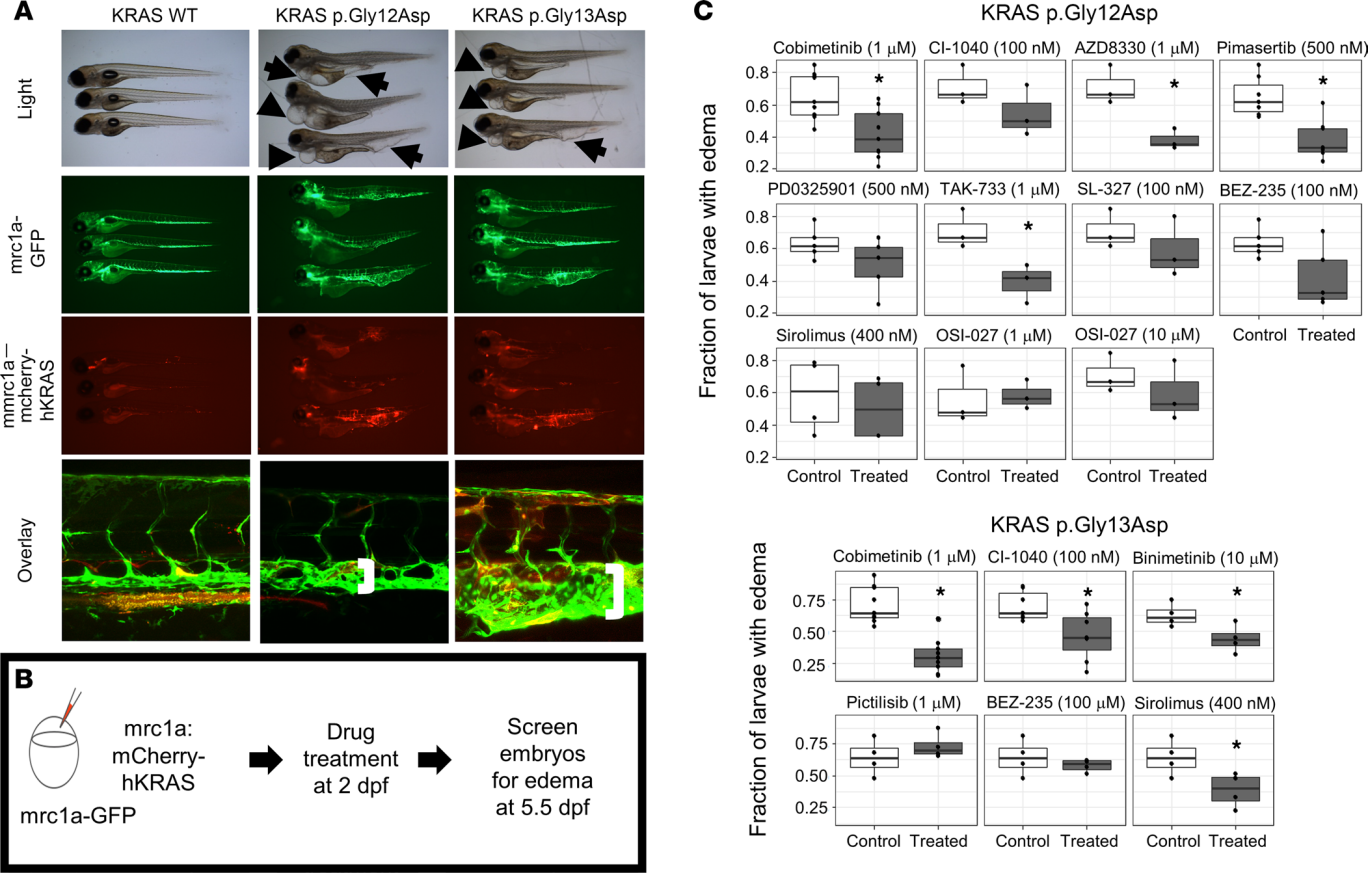
In vivo zebrafish larvae modeling and therapeutic screening. (**A**) Embryos were injected at 0-cell stage with either mrc1a:wt-hKRAS, mrc1a:hKRAS p.Gly12Asp, mrc1a:hKRAS p.Gly13Asp and tol2 transposase. Larvae at 7 dpf under light microscopy (2.5× magnification), GFP (2.5× magnification), mCherry (2.5× magnification), and confocal microscopy (20× magnification). Larvae injected with mrc1a:hKRAS p.Gly12Asp or p.Gly13Asp under light microscopy have edema around the heart and intestine (arrows). Mrc1a:wt-hKRAS have essentially normal vasculature of larvae injected with under confocal microscopy. Vasculature of larvae injected with mrc1a:hKRASp.Gly12Asp or mrc1a:hKRASp.Gly13Asp under confocal microscopy showing fusion of the thoracic duct with the cardinal vein (brackets). (**B**) In vivo zebrafish larvae therapeutic screening. Embryos were treated at 48 hpf and screened for edema at 5.5 dpf. (**C**) Fraction of larvae with edema by *KRAS* variant, drug, and concentration. Each dot represents a single experiment. *P < 0.05 by unpaired, 1-tailed Student’s *t* tests, after correction for multiple testing with the Benjamini and Hochberg FDR method. The mechanism of action for each drug can be seen in Supplemental Table 1. Due to figure legend space limitations, the number of zebrafish larvae for each experiment are in Supplemental Table 2.

We used a transient transgenic model, which leads to mosaic expression, to simulate the mosaicism seen in the study individuals. Human WT KRAS, KRAS p.Gly12Asp, or KRAS p.Gly13Asp were transiently expressed under the *mrc1a* promoter to drive expression in venous and lymphatic endothelium. These proteins were combined with mCherry (a red fluorescent protein), which is a marker of transgenesis. The models were performed in a stable transgenic zebrafish line *Tg(mrc1a:gfp*), which expressed GFP in the venous and lymphatic endothelium. There was no detectable effect with WT KRAS expression (Figure 4 and Supplemental Figure 4), while larvae expressing KRAS p.Gly13Asp had a significant increase in the fraction of larvae with edema at 5 dpf (*P* = 1.76 × 10^–4^; Supplemental Figure 4), showing that edema was caused by mutant KRAS and not the injection procedure and demonstrating WT KRAS as an adequate control. Larvae with mosaic, transient expression of KRAS p.Gly12Asp or p.Gly13Asp (represented by mCherry) showed edema around the heart, fusion of the thoracic duct with cardinal vein, and tumor-like expansion of lymphatic tissue (Figure 4A). Although the mosaicism varies, the effect is similar, suggesting that there may be an effect on the surrounding cells or that perhaps different degrees of lymphatic dysfunction may result in the same phenotype. These results showed striking similarities with the findings on individual 1’s MRL (Figure 1).

Given that the zebrafish larvae transiently expressing the mutant KRAS modeled the lymphatic dysplasia, we next sought to perform preclinical studies to identify molecularly targeted therapy as was performed in the cellular models. We hypothesized that inhibitors against downstream effectors of RAS/MAPK signaling would help prevent the development of lymphatic anomalies better than sirolimus, an mTOR inhibitor that is downstream of PI3K signaling (Supplemental Table 1). *Tg(mrc1a:gfp)* embryos were injected with an activating *KRAS* variant construct, treated from 2–5.5 dpf with a pharmacologic agent, and screened for rescue of edema as a metric of improvement (Figure 4B). MEK inhibition reduced the fraction of larvae expressing KRAS p.Gly12Asp with edema, but not all inhibitors led to a statistically significant reduction in the dose range used. Specifically, MEK inhibition with cobimetinib (1 μm), AZD8330 (1 μm), pimasertib (500 nm), or TAK-733 (1 μm) significantly reduced the fraction of larvae with edema (*P* = 0.0113, *P* = 0.030, *P* = 0.00765, and *P* = 0.04, respectively); however, MEK inhibition with CI-1040 (0.1 μm), PD0352901 (500 nm), or SL-327 (0.1 μm) (*P* = 0.178, *P* = 0.129, and *P* = 0.262, respectively) did not (Figure 4C). Interestingly, combined PI3K and mTOR inhibition with BEZ-235 (100 nm) also reduced the fraction of larvae expressing KRAS p.Gly12Asp with edema, although after correction for multiple tests, this was not significant (*P* = 0.051) (Figure 4C). mTOR inhibition with sirolimus (400 nm) or OSI-027 (1 μm or 10 μm) did not significantly improve the fraction of larvae expressing KRAS p.Gly12Asp with edema (Figure 4C; *P* = 0.30, *P* = 0.44, and *P* = 0.44). In the *KRAS* p.Gly13Asp zebrafish model, MEK inhibition with cobimetinib (1 μm), CI-1040 (100 nm), and binimetinib (1 μm) all significantly reduced the fraction of larvae with edema compared with the control group (Figure 4C; *P* = 1.61 × 10^–4^, *P* = 0.031, and *P* = 0.040). In contrast to the p.Gly12Asp model, sirolimus treatment resulted in a significant reduction of the fraction of larvae with edema in the p.Gly13Asp model (Figure 4C; *P* = 0.040). Neither combined PI3K and mTOR inhibition with BEZ-235 (100 nm) nor pan-PI3K inhibition with pictilisib (1 μm) led to a significant reduction of the fraction of larvae with edema (Figure 4C; *P* = 0.262 and *P* = 0.233).

### Treatment.

Based on these studies, and prior experiences of successful treatment of CCLA with trametinib, individuals 2 and 4 were initiated on trametinib therapy. Dosing was based on the experience in pediatric oncology patients with a goal of 0.025 mg/kg once daily. Individual 2 is currently treated with trametinib as well as weaning doses of sirolimus (0.3 mg twice daily of sirolimus). Individual 4 started on low dose, 0.005 mg/kg, rounded to the nearest tablet size of trametinib of 0.5 mg by mouth daily. Trametinib was discontinued due to an episode of rhabdomyolysis. Rhabdomyolysis is an adverse effect of trametinib that has been seen in less than 10% of participants in the METRIC study, less than 10% of participants in the COMBI-d study (combined with dabrafenib, a BRAF inhibitor), and less than 1% of participants in the *COMBI-AD* Study (31).

## Discussion

CLAs present a vastly understudied disease category with limited treatment options. In this study, we verify mosaic, activating *KRAS* pathogenic variants as a cause of CCLA and lymphedema and report what we believe is the first microcystic lymphatic malformation caused by mosaic, activating *KRAS* pathogenic variants (4, 32). We defined the lymphatic anomalies in 4 individuals with mosaic, activating *KRAS* pathogenic variants, 3 with epidermal nevus syndromes and 1 without. Initial reports suggested that ECCL, OES, and NSS were different syndromes; however, others have recently suggested these syndromes should be considered a spectrum and proposed the term “mosaic RASopathies” (13, 33). There has been no prior report of phenotypic overlap between the syndromes like individual 1’s phenotype of both ECCL and NSS. The osseous thinning of the skull (34), polycystic kidneys (35), multifocal tachycardia (36, 37), hemimegalencephaly (38), and coarctation of the aorta (37) are all seen in our cohort. The individuals we report here strengthen the association of these findings with the phenotype of mosaic RASopathies.

The individuals in this study highlight the potentially novel variety of lymphatic anomalies that may be seen in ECCL, NSS, or OES. Lymphatic anomalies have rarely been reported with ECCL (34), though the association with lymphatic anomalies and germline RASopathies is well established (4, 39–41). Additionally, activating *KRAS* pathogenic variants have also recently been described as a cause of aberrant vascular development, vascular malformation, and lymphatic anomalies including lymphedema, chylothorax, macrocystic lymphatic malformation, lymphangiectasia, and Gorham-Stout disease (10–13, 34, 40, 42–44).

The lymphangiogram in individuals 1, 2, and 4 demonstrated extensive central conducting lymphatic anomalies, which have rarely been reported in *KRAS*-related disorders (4). However, symptoms of CLAs have been described in patients with mosaic KRASopathies, including limited natural history data in 3 individuals reported by Chang and colleagues (13). Patient 4 in the case series underwent lymphatic embolization for recurrent chylothorax (specific vessel and embolization material are not noted). Patient 7 was born after a pregnancy complicated by hydrops. Postnatally, he developed persistent chylothorax and died at 3 weeks of age. Autopsy revealed lymphangiectasia of the neck and scalp. Patient 8 in the case series was born after a pregnancy complicated by fetal hydrops and in utero chylothorax requiring bilateral chest shunts. The patient also had T cell immunodeficiency, which can be a result of intestinal lymphangiectasia and resulting protein-losing enteropathy. Future studies to evaluate the natural history of disease will assist in prognostication for patient outcomes.

Interestingly, not all individuals with activating *KRAS* variants have lymphatic dysplasia (38, 45). One possible explanation is that the degree of lymphatic dysplasia is affected by the distribution of the activating *KRAS* variant, as has previously been proposed for *PIK3CA*-related overgrowth spectrum disorders (46, 47). Another possible explanation is that some variants are more activating compared with others, as observed in our studies. Further work is needed to understand if these variants are present in the lymphatic endothelium and if this is a cell-autonomous or -nonautonomous phenomenon.

RAS proteins (especially KRAS) are important for lymphatic development, which is thought to be due to modulation of VEGFR3 (48). Future work should examine the effects of VEGF-C stimulation in these models. The molecular studies performed in this work demonstrate that *KRAS* variants activate MAPK signaling and suggest crosstalk to the PI3K/AKT/mTOR pathway. Using in vitro and in vivo modeling, we demonstrated that downregulating MAPK activity using MEK inhibitors improves the lymphatic phenotype as measured by the decreased fraction of larvae with edema or decreased cumulative sprout length and the number of sprouts per sphere. It is important to note as a limitation of this study that MEK inhibition did not completely rescue the phenotype in the zebrafish model.

Further work is needed to understand the mechanism by which MEK inhibition leads to improvement in the patients. We hypothesize that restoration of the normal level of signaling may improve endothelial cell junction integrity as demonstrated in our 2D model. Trametinib may also improve the function of lymphatic valves, as has been demonstrated in the KRAS p.Gly12Asp mouse model. Previous work by our group demonstrates decreased retrograde flow and symptomatic improvement after MEK inhibitor therapy in adolescent patients with established disease caused by pathogenic variants causing activation of the RAS pathway (20–23). Nonetheless, in combination with previous work in a *KRAS-*driven mouse model of Gorham-Stout disease, our data suggest that use of a MEK inhibitor for the treatment of lymphatic anomalies due to pathogenic variants in the RAS/MAPK pathway should be systematically evaluated in future trials (12, 20–23).

Although mTOR inhibition did not lead to a statistically significant improvement in our in vivo *KRAS* p.Gly12Asp model, it did in the p.Gly13Asp model. Interestingly, 2 of the individuals in this study reported improvement with sirolimus. Individual 2 entered a period of stability before developing worsening disease and addition of other agents, and individual 4 reported improvement in his scrotal chylous leak. Additionally, improvement with sirolimus has been reported for an individual with lymphangiectasia and overgrowth due to *KRAS* p.Gly12Asp, though others have reported no improvement (9, 12). Similar to individual 2, combinatorial therapy has previously been reported (39).

Interestingly, combined inhibition of the PI3K pathway by BEZ-235 treatment led to a nonsignificant reduction in edema, greater in the p.Gly12Asp compared with the p.Gly13Asp zebrafish model. Moreover, phosphorylation of S6 was elevated in the p.Gly13Asp spheroid model and treatment with MEK inhibition modulates S6 phosphorylation, suggesting there is activation of upstream proteins. Further nuances of context-dependent alterations in molecular signaling may help elucidate the different responses.

Activation of the PI3K/AKT/mTOR pathway has been demonstrated in multiple KRAS-positive cancers. Human Wilms’ tumors harboring the KRAS p.Gly12Asp pathogenic variant and not the 14 most common *PI3KCA* pathogenic variants demonstrated increased pAKT staining on tissue array (49). In *KRAS* mutant colorectal cancer lines with combined EGFR and MEK inhibitor resistance, compensatory PI3K/AKT signaling plays an important escape mechanism (50). In addition, in a *KRAS* metastatic colorectal cancer model, PI3K inhibitor BKM120 resulted in decreased tumor volume (51). Interestingly, in a *KRAS-*driven mouse model of lung adenocarcinoma combinatorial therapy with NVP-BEZ235 and ARRY-142886, a MEK inhibitor resulted in a larger reduction of tumor volume compared with NVP-BEZ235 alone (52). Further preclinical studies and possible systematic trials are needed to evaluate the efficacy of combined MEK and mTOR inhibition as has been shown for pancreatic cancer and understand the mechanism (53).

We and others have modeled variants to establish appropriate therapeutics for individuals with vascular malformations (12, 20, 44, 54, 55). The in vitro and in vivo systems described here can be scaled to screen additional suspected gain of function variants for pathogenicity. They may also be used in preclinical therapeutic studies for individualized therapy, as we have done. Additionally, the doses used in these systems provide some evidence for the dosage ranges to be used in future clinical trials.

In summary, we defined the lymphatic dysplasia in 4 individuals with pathogenic, mosaic, activating *KRAS* variants. In these studies, we used in vitro and in vivo modeling in a precision medicine approach to identify a molecularly targeted therapy for the treatment of lymphatic disorders. Long-term follow-up is needed to understand the efficacy of MEK inhibition in the individuals presented here. These studies are the foundation for future clinical trials to evaluate the utility of MEK inhibitors for treatment of CCLA due to genetic pathogenic variants that activate the RAS/MAPK pathway in humans.

## Methods

### Individuals

Individual 1 had clinical genetic testing performed at Genomics and Pathology Services at Washington University School of Medicine. Individual 1 (including a different sequence from his DCMRL) is included in a cohort study examining genetics of CCLA (4). Individual 2 had clinical genetic testing performed at GeneDX. Individual 3 had genetic studies performed as previously published (9). Individual 4 had clinical genetic testing performed at the University of Pennsylvania Genetic Diagnostic Laboratory.

### HDLEC methods

#### Expression and characterization of KRAS4A pathogenic variant in primary HDLECs.

HEK293T cells were obtained from the American Type Culture Collection and grown at 37°C in DMEM supplemented with 10% FBS. Primary juvenile HDLECs were obtained from Promocell and were cultured in Endothelial Cell Growth Medium MV 2 (Promocell) according to the manufacturer’s directions. The *KRAS4A* sequence, corresponding to the National Center for Biotechnology Information Reference Sequence (NM_033360.4), was amplified from a cDNA library, subcloned into pBabe-Puro-CMV^+^ (21) with an N-terminal FLAG tag, and confirmed by Sanger sequencing. Viral production was performed using FugeneHD (Promega), with 8 μg of total DNA (KRAS expression plasmid together with envelope and packaging plasmids) and 18 μL of the transfection reagent in HEK293T. After 48 hours, viral supernatant was diluted in half with HEK293T culture media, supplemented with 8 μg/mL Polybrene (MilliporeSigma), and filtered through a 0.45 μm filter (VWR). HDLECs were spinfected at 650*g* for 90 minutes, and subsequently cultured for 6 hours, at which point the viral supernatant was replaced by standard endothelial cell culture medium. Transduced HDLECs were cultured for 48 hours before use in experiments.

### Determination of KRAS4A expression via qPCR

RNA was extracted from transduced HDLECs using RNeasy Mini Kit with on-column DNase digestion (QIAGEN). Conversion to cDNA was performed using 2 μg of RNA with the High Capacity cDNA Reverse Transcriptase Kit (Thermo Fisher Scientific). qPCR was performed using primers for *KRAS* (TCTTGGATATTCTCGACACAGC and TAATTTTCACACAGCCAGGAGT) and *GAPDH* (ATCAGCAATGCCTCCTGCAC and TGGCATGGACTGTGGTCATG). PCR was performed and data were acquired on a Viia7 qPCR machine (Thermo Fisher Scientific) using triplicate wells. Data were analyzed in Quantstudio Real-Time PCR Software v1.3 using *GAPDH* as the normalization control, normalized to the expression of *KRAS* WT. Results were exported as relative quantities and 95% CIs and plotted using R 4.1.3.

### Immunofluorescence staining of HDLECs

Immunofluorescence staining of HDLECs was performed as previously described (20). Briefly, transduced HDLECs were plated onto gelatin-coated coverslips in the presence or absence of inhibitors and cultured for 48 hours. Cells were washed and then fixed in paraformaldehyde. Fixed cells were washed twice with PBS and twice with 0.1% BSA in PBS. Cells were permeabilized and blocked by incubation with 10% normal donkey serum (Jackson ImmunoResearch) and 0.3% Triton X-100 (Sigma-Aldrich) in PBS. VE-cadherin polyclonal antibody (Thermo Fisher Scientific, catalog 36-1900) was diluted (final concentration 2 mg/mL) in 0.01% normal donkey serum, 0.1% BSA, and 0.3% Triton X-100 in PBS, and staining was performed for 1 hour. Coverslips were washed twice with 0.1% BSA in PBS. Goat anti-rabbit Alexa Fluor 546 (Thermo Fisher Scientific, catalog A11010; final concentration 8 mg/mL) and phalloidin Alexa Fluor 647 (Thermo Fisher Scientific, catalog A22287; final concentration 5 U/mL) were diluted in 0.01% normal donkey serum, 0.1% BSA, and 0.3% Triton X-100 in PBS, and staining was performed for 1 hour. Coverslips were washed twice with 0.1% BSA in PBS and twice with PBS. Coverslips were mounted to slides using Prolong Gold antifade reagent (Thermo Fisher Scientific). Image acquisition was performed on a Leica DM6000 motorized upright microscope with a Photometrics HQ2 high-resolution monochrome charge-coupled device camera using LAS AF software (Leica Microsystems). *Z*-stacks were acquired at 10× original magnification. Images were further processed in the Fiji software package (56). Brightness and contrast adjustments were made. Identical brightness and contrast settings were applied to all images.

### Sprouting assay

Multicellular spheroids for the lymphatic sprouting assay were formed by plating 5,000 HDLECs expressing KRAS WT or mutants into wells of a 96-well plate that were precoated with 1.5% agarose. Under these conditions, the HDLECs would aggregate into a spheroid within 24 hours. After formation, each spheroid was transferred into a gelling solution comprised of type I collagen (Corning, catalog 354236; final concentration 1.5 mg/mL; pH neutralized with NaOH) and inhibitors at the indicated concentrations, which was then allowed to polymerize at 37°C. Once solidified, Endothelial Cell Growth Medium MV 2 containing inhibitors at the appropriate concentration was added onto the collagen gels. After 1 day of incubation, *Z*-stack images with a step size of approximately 8.5 μm were taken of the embedded spheroids using an EVOS FL Auto Imaging System (Thermo Fisher Scientific) at 4× original magnification. The numbers and lengths of capillary-like sprouts growing from each spheroid were measured with Fiji using the NeuronJ plugin (57).

### Biochemistry with spheroids

To obtain protein extracts from HDLECs spheroids, 8 spheroids (40,000 cells total) were embedded in the same well with inhibitor or vehicle to increase the ratio of cellular protein to collagen. Embedded spheroids were cultured for 24 hours, at which point the collagen gel was scraped from the plate, transferred to a microfuge tube, and homogenized in RIPA buffer (Roche; 50 mM Tris pH 7.4, 150 mM NaCl, 1% Nonidet P-40, 0.5% sodium deoxycholate, 0.1% SDS, 1 mM sodium orthovanadate, 50 mM sodium fluoride, and protease inhibitors). Lysates were clarified by centrifugation at 20,000g, at 4°C, for 10 minutes and analyzed by SDS-PAGE and Western blotting with phospho-S6 ribosomal protein (pS6; Cell Signaling Technology, [Ser240/244] [D68F8] XP Rabbit mAb 5364, clone D68F8); pERK (Cell Signaling Technology, Phospho-p44/42 MAPK [Erk1/2] [Thr202/Tyr204] Ab 9101, Polyclonal); and β-actin (Santa Cruz Biotechnology, sc-69879, clone AC15).

#### HDLEC drug treatment and 2D biochemistry.

Procedures were as previously described (21). Abs used for 2D biochemistry were pS6 Ribosomal Protein (Cell Signaling Technology [Ser240/244] [D68F8] XP Rabbit mAb 5364, clone D68F8); pERK (Cell Signaling Technology, Phospho-p44/42 MAPK [Erk1/2][Thr202/Tyr204] Ab 9101, Polyclonal); and β-actin (Santa Cruz Biotechnology, sc-69879, clone AC15). Blots in Supplemental Figure 2 additionally used were S6 Ribosomal Protein (Cell Signaling Technology, [54D2] Mouse mAb 2317, clone 54D2); ERK1/2 (Cell Signaling Technology, p44/42 MAPK [Erk1/2] [L34F12] Mouse mAb 4696, clone L34F12); pAKT S473 (Cell Signaling Technology, [Ser473] [D9E] XP Rabbit mAb 4060, clone D9E); Akt (Cell Signaling Technology, [pan] [C67E7] Rabbit mAb 4691, clone C67E7); and GAPDH (Santa Cruz Biotechnology, sc-47724, clone 0411]).

### Zebrafish methods

*Tg(mrc1a:GFP)* in a *casper* (*nacre^–/–^ roy^–/–^*) background zebrafish were a gift from Brant Weinstein’s laboratory (*Eunice Kennedy Shriver* National Institute for Child Health and Human Development, Bethesda, Maryland, USA).

#### Transgenic expression of human KRAS in zebrafish.

Human WT *KRAS4B*, *KRAS* p.Gly12Asp, and *KRAS* p.Gly13Asp cDNAs were subcloned into the N-terminal multisite gateway vector p3E. Expression constructs were assembled using a Tol2 backbone vector including a gateway cloning cassette with the *mrc1a* promoter, followed by mCherry and then *KRAS* in the same reading frame. This orientation was chosen because *KRAS* has a CAAX box at the end for membrane localization and KRAS protein is not functional if a protein is attached at the 3′ end. Constructs were coinjected with *Tol2* mRNA in *Tg(mrc1a:GFP)* zebrafish. The zebrafish *mrc1a* promoter drives expression in the vein and lymphatic endothelium (26). To avoid pigmentation, zebrafish were maintained in a *casper* (*nacre^–/–^ roy^–/–^*) background (58). For imaging, larvae were either photographed in E3 medium using a dissecting scope or mounted in low-melting agarose, and multiple *Z*-images were taken with a Zeiss LSM710 confocal microscope using a 20× lens. Confocal *Z*-stacks of images were superimposed using Zeiss Zen software’s maximum intensity projection function and the lymphatic structure was analyzed. Three independent experiments were performed with approximately 6–8 larvae analyzed per experiment. Sexual determination of zebrafish larvae had not yet occurred at the time points used in this study.

#### Inhibitory drug treatment in zebrafish.

Pharmacologic treatments were performed in 6-well plates. Expression construct injected larvae were treated starting at 48 hours. Media and reagents were replaced every 24 hours. Pharmacologic agents were diluted in embryo medium containing 0.01 M Tris pH 7.2 and 0.1% DMSO. Lymphatic defects in the activating KRAS mutants caused the formation of large edematous areas around the intestine and heart at 5 days after fertilization. Larvae were scored for the reversal of the edema phenotype.

Doses for zebrafish were based on previous literature or titration experiments. Typically, we used doses at half maximal inhibitory concentration from the known pharmacokinetic profile and doses 1 and 2 logs above and below that to determine the therapeutic range and evaluate for any developmental defects.

### Statistics

For all the assays performed on HDLECs, at least 3 independent experiments were performed with independent transductions of HDLECs and 1-sided Student’s *t* tests were performed to calculate significance from Western blotting. For spheroid assays, 2-sided Student’s *t* tests were performed to calculate significance. Comparisons were made between DMSO-treated WT and both DMSO-treated mutants, as well as each DMSO-treated mutant and all the drug treatments of that mutant; *P* values were corrected for multiple testing with the Benjamini and Hochberg FDR method. All the zebrafish-related assays were performed in at least 3 independent experiments and tested by unpaired, 1-tailed Student’s *t* tests for comparison of 2 groups; *P* values were corrected for multiple testing with the Benjamini and Hochberg FDR method. *P* values less than 0.05 were considered significant.

### Study approval

Individuals 1, 2, and 4 were consented into an IRB-approved protocol (16-013278) held by the Center for Applied Genomics and provided informed consent for photo publication. Individual 3 provided informed consent for photo publication and provided written informed consent before participation in this study. All procedures using zebrafish were approved by the IACUC of Children’s Hospital of Philadelphia (IAC 001154) and were in accordance with the *Guide for the Care and Use of Laboratory Animals* (National Academies Press, 2011).

## Author contributions

SES, MEM, CS, and HH conceived and designed the study. SES, MEM, CS, LSM, SEK, AIR, NK, ES, EP, IJF, TH, JTS, IDK, LCS, KS, YD, NR, JRCP, RN, VRS, AG, CLS, JCG, PL, DMB, NO, CH, DS, and TAN collected the data. SES, MEM, CS, DL, MRB, CK, CLS, and HH analyzed the data. LFS collected and analyzed the data. SES, MEM, CS, and CLS wrote the manuscript. All authors edited and approved the manuscript. SES, DL, and HH provided funding.

## Supplementary Material

Supplemental data

## Figures and Tables

**Table 1:**
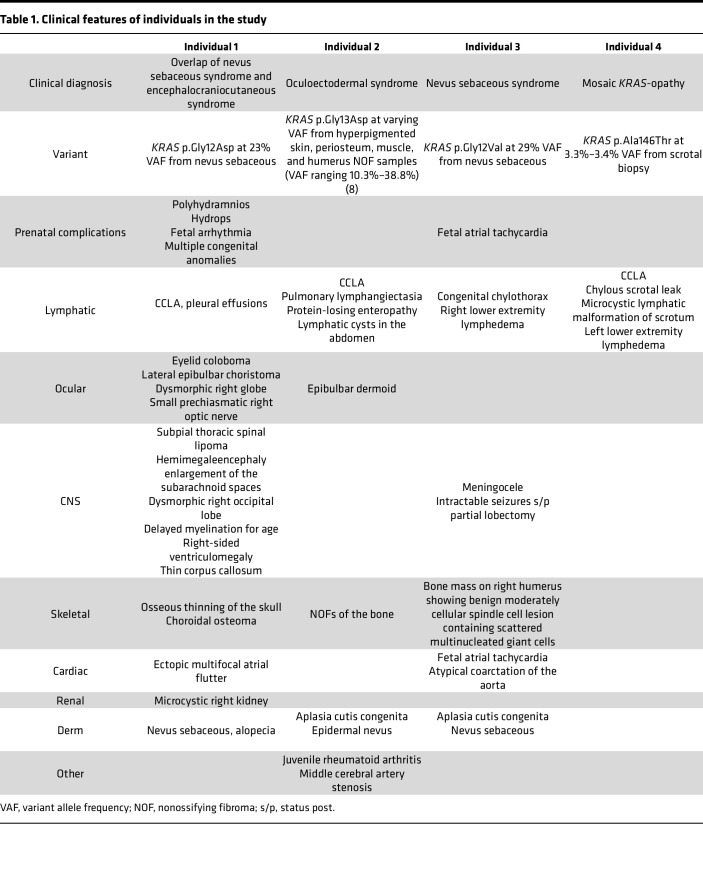
Clinical features of individuals in the study

## Version 1 05/08/2023

Electronic publication

## Version 2 05/24/2023

There was a mislabeled value in Figure 4C. It is now corrected
