# Supplementary material for: Lymphatic disorders caused by mosaic, activating KRAS variants respond to MEK inhibition
Source: JCI Insight. 2023 May 8;8(9):e155888. doi: 10.1172/jci.insight.155888 (PMC10243805; doi:10.1172/jci.insight.155888)
Supplement: Supplemental data [file jciinsight-8-155888-s241.pdf]

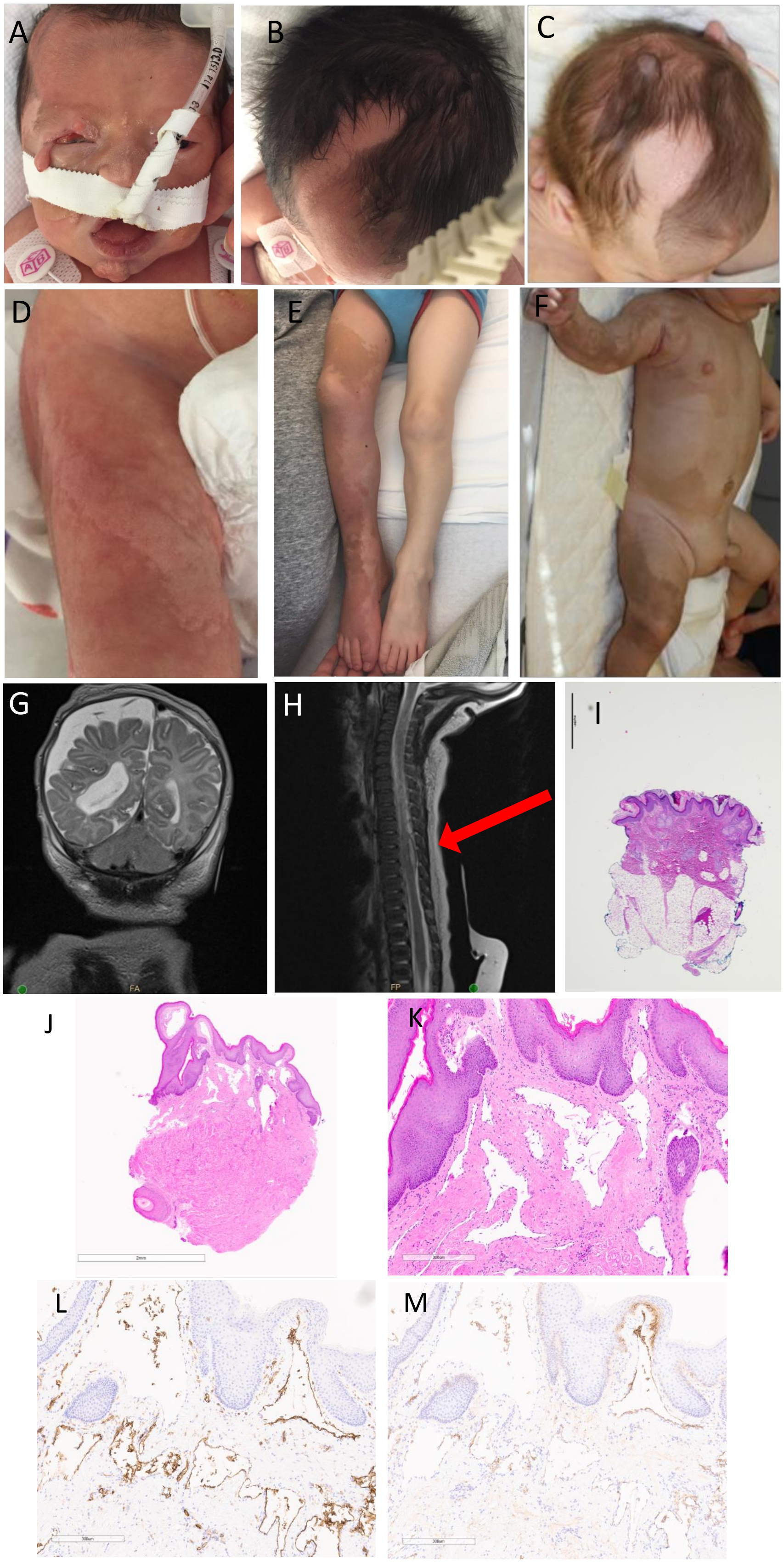
A
B
C
D
E
F
G
H
I
J
K
L
M

Supplemental Figure 1: Participant photographs, imaging, and pathology. A. Eyelid coloboma, a lateral epibulbar choristoma, a pedunculated papule on the eyelid, nevus sebaceous on midline face (Individual 1 at 1 day old). B. Nevus psiloparus on scalp (Individual 1 at 1 day old). C. Nevus sebaceous and cutis aplasia (Individual 3 at 4 months old). D. Nevus sebaceous on leg (Individual 1). E. Hemihypertrophy and edema of right leg (Individual 3 at 4 years old). F. Extensive nevus sebaceous following blashkoid lines (Individual 3 at 4 months old). G. Brain MRI demonstrates enlargement of the extra-axial spaces overlying the right frontal, parietal and temporal lobes and asymmetric volume loss in the right cerebral hemisphere, including the frontal lobe, parietal lobe, temporal lobe and occipital lobe, with associated ventriculomegaly & deep sulci (Individual 1). H. Spine MRI demonstrates extramedullary, intraspinal lipoma measuring approximately 6 mm AP x 6 mm transverse x 2.5 cm craniocaudal along the dorsal surface of the thoracic spinal cord from approximately T5-6 down to T9 (Individual 1). I. Histology of nevus sebaceous at 10x magnification. The epidermis shows papillomatosis and hyperkeratosis. The dermis shows a few sebaceous lobules. The specimen contains a sparse inflammatory infiltrate (Individual 1). J. Low power view of lymphatic malformation with subepithelial microcystic channels (Individual 4, H&E, 20x). K. Higher power of microcystic lymphatic changes with flattened endothelial cells (Individual 4, H&E, 65x). L. CD31 staining of lymphatic endothelial cells (Individual 4, 65x). M. D2-40 staining of lymphatic endothelial cells (Individual 4, 65x).

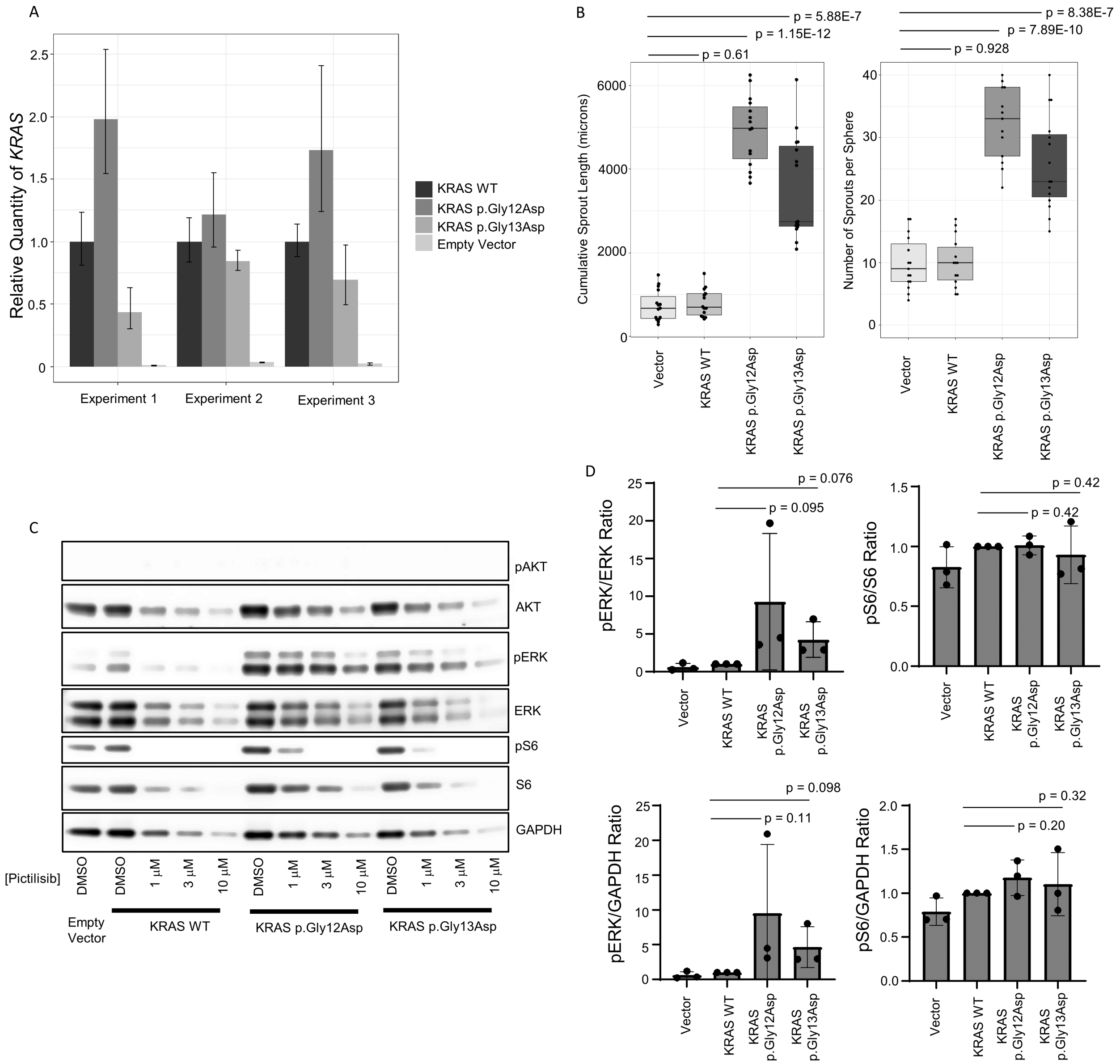

Supplemental Figure 2: Expression of *KRAS* WT, p.Gly12Asp, and p.Gly13Asp in HDLECs. A. Expression of retrovirally transduced KRAS was determined by qRT-PCR using primers targeting *KRAS* and *GAPDH*. Expression was normalized to *GAPDH* and to expression of *KRAS* WT. The error bars in the qPCR data stretch from RQ-Min to RQ-Max, as calculated by the Quantstudio Software using the intra-experiment sample replicates. They represent the 95% confidence interval for the expression range. B. Spheroid sprouting assays were performed with HDLECs transduced with KRAS variants or empty retroviral vector. Data were quantitated from 3 independent experiments showing cumulative sprout length per sphere (left) and number of sprouts per sphere (right). In the box and whisker plots, the center line is the median, the lower and upper boundaries of the box are the 25% and 75% quartiles, and the whiskers extend to 1.5-times the interquartile range from the 25% and 75% quartiles. C. Cell lysates from HDLECs transduced with *KRAS* variants were immunoblotted for pERK at T202 and Y204, pS6 at S235/236, and pAKT at S473. Blots were reprobed for total ERK, S6, and AKT, and finally reprobed for GAPDH. D. Quantification of immunoblotting of the DMSO-treated condition from 3 independent experiments, pERK or pS6 normalized to either total protein (ERK or S6) or GAPDH, normalized to WT + DMSO sample. Bars are means, error bars are standard deviations. 1-sided student t-tests were performed to calculate significance, and p-values were adjusted for multiple testing using the FDR (Benjamini and Hochberg) method.

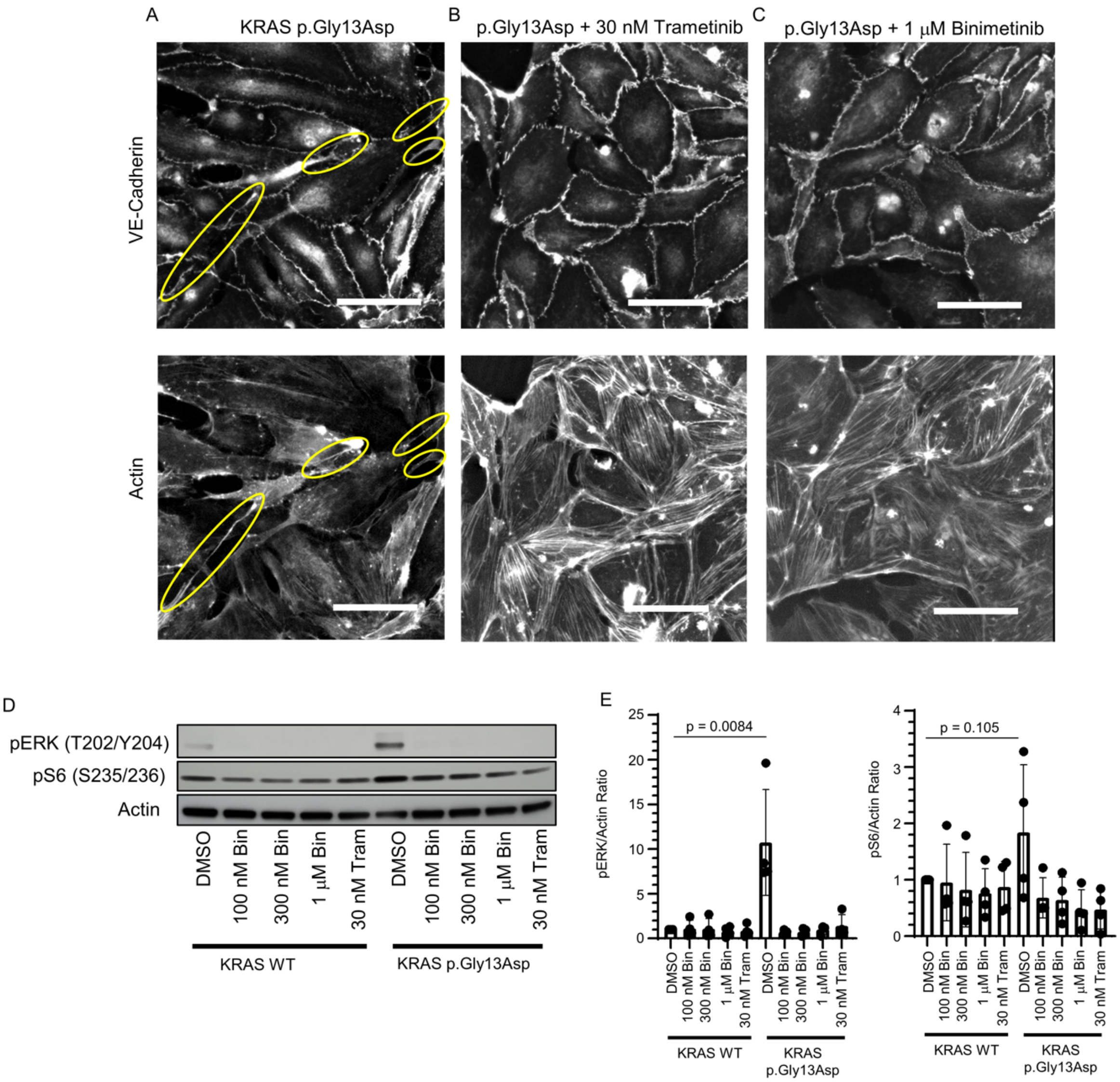

Supplemental Figure 3: Expression of *KRAS* p.Gly13Asp in HDLECs. A. Human dermal lymphatic endothelial cells (HDLECs) expressing KRAS p.Gly13Asp were stained with VE-cadherin or actin. Yellow circles show extensions from cells. B. KRAS p.Gly13Asp treated with 30 nm trametinib. C. KRAS p.Gly13Asp treated with 1 mM binimetinib. Scale bars in all images represent 50 microns. D. Cell lysates from HDLECs transduced with either KRAS WT or p.Gly12Asp were immunoblotted for pERK at T202 and Y204 or pS6 at S235/236 with actin as control. Data were quantitated and from 4 independent experiments. E. Quantification of immunoblotting from 4 independent experiments, pERK or pS6 normalized to actin, normalized to WT + DMSO sample. Bars are means, error bars are standard deviations. Bin = binimetinib. Tram = trametinib. 1-sided student t-tests were performed to calculate significance.

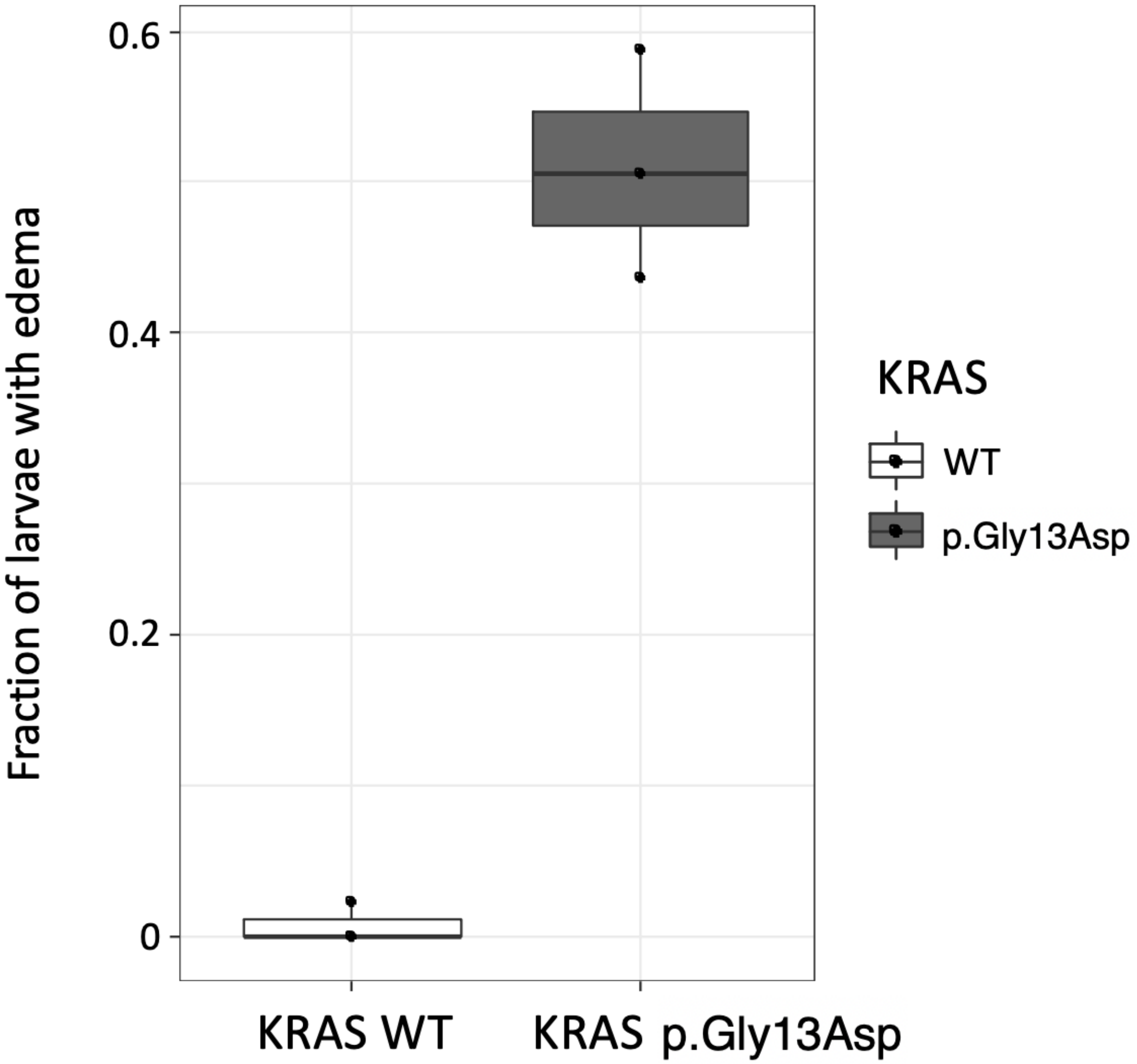

Supplemental Figure 4: Quantification of edema in mrc1a:wt-hKRAS (total n =130) and mrc1a:hKRAS p.Gly13Asp (total n = 166) larvae at 5 dpf from three independent experiments. P = 0.0001 by unpaired, one-tailed Student's *t*-tests.

Supplemental Table 1: Pharmaceutical compounds trialed or mentioned in this study

Drug Name	Target	Mechanism of Action	IC50	Current Developer	Development Stage	Indications
trametinib	MEK1, MEK2	ATP-non-competitive, allosteric inhibitor	0.92nM, 1.8nM	Novartis	FDA approved	Metastatic melanoma, lung cancer, thyroid cancer with BRAF V600E/V600K mutation
cobimetinib	MEK1	ATP-non-competitive, allosteric inhibitor	4.2nM	Exelixis, Roche	FDA approved	Metastatic melanoma with BRAF V600E/V600K mutation in combination with vemurafinib
binimetinib	MEK1/2	ATP-non-competitive, allosteric inhibitor	12nM	Pfizer, Array BioPharma	FDA approved	Unresectable or metastatic melanoma in combination with BRAF V600E/V600K mutation in combination with encorafinib
ulixertinib	ERK1, ERK2	ATP-competitive inhibitor	<0.3nM	Biomed Valley Discoveries	Phase II	Multiple cancers
dactolisib (BEZ235)	Class I PI3K (p110α, p110γ, p110δ, p110β) mTOR, ATR	ATP-competitive inhibitor	4nM, 5nM, 7nM, 6nM, 21nM	resTORbio, Novartis	Phase III	Respiratory tract infections, multiple cancers
Pictilisib (GDC-0941)	Class I PI3K (p110α, p110γ, p110δ, p110β)	ATP-competitive inhibitor	3 nm	Genentech (prior to termination)	Phase II	Breast cancer, lung carcinoma
pimasertib	MEK1/2	ATP-non-competitive, allosteric inhibitor	5nM-2µM	Day One Pharmaceuticals, Merck KGaA, Sanofi	Phase II	Multiple cancers
AZD8330	MEK1/2	ATP-non-competitive inhibitor	7nM	Astrazeneca	Phase I	Multiple cancers
TAK-733	MEK1	ATP-non-competitive, allosteric inhibitor	3.2nM	Takeda	Phase I	Multiple cancers
SL-327	MEK1, MEK2	Unknown	180nM, 220nM	N/A	Pre-clinical	N/A
mirdametinib (PD032901)	MEK1/2	ATP-non-competitive, allosteric inhibitor	0.33nM	Springworks Therapeutics	Phase II	Neurofibromatosis, multiple cancers
CI-1040 (PD184352)	MEK1/2	ATP-non-competitive, allosteric inhibitor	17nM	Pfizer	Phase II (development abandoned)	Multiple cancers
PD0325901	MEK	selective and non ATP-competitive inhibitor	0.33 nM	SpringWorks Therapeutics, Inc	Phase II	Plexiform neurofibroma Neurofibromatosis Type 1
Sirolimus (Rapamycin)	FKBP-12	Complex with FKBP-12 binds and	0.1nM	Pfizer	FDA approved for non-lymphatic	Organ transplant rejection, lymphangioleiomyomatosis

		inhibits mTOR			disorder indications	
OSI-027 (CERC-006)	mTORC1, mTORC2	ATP-competitive inhibitor	22nM, 65nM	Cerecor	Phase II	Lymphatic malformation, renal cancer

Supplemental Table 2: Experimental numbers for Figure 4. Cobimetinib was used as a control for experiments, thus the number of experiments is much greater than the others.

**Variant**	**Drug**	**Number of experiments**	**Control (total n)**	**Variant (total n)**	**p-value**	**FDR-adjusted p-value**
p.Gly12Asp	Cobimetinib	9	248	271	0.002	0.0113
p.Gly12Asp	CI-1040	3	47	61	0.115	0.178
p.Gly12Asp	AZD8330	3	47	51	0.007	0.030
p.Gly12Asp	Pimasertib	7	177	198	0.0009	0.00765
p.Gly12Asp	PD0325901	5	151	151	0.076	0.129
p.Gly12Asp	TAK-733	3	47	51	0.016	0.040
p.Gly12Asp	SL-327	3	47	55	0.204	0.262
p.Gly12Asp	BEZ-235	5	145	135	0.027	0.051
p.Gly12Asp	Sirolimus	4	161	142	0.303	0.343
p.Gly12Asp	OSI-027 (1µM)	3	105	97	0.438	0.438
p.Gly12Asp	OSI-027 (10µM)	4	161	163	0.435	0.438
p.Gly13Asp	Cobimetinib	9	224	213	9.46×10^{-6}	1.61×10^{-4}
p.Gly13Asp	CI-1040	7	172	177	0.009	0.031
p.Gly13Asp	Binimetinib	4	107	103	0.018	0.040
p.Gly13Asp	Pictilisib	4	123	126	0.165	0.233
p.Gly13Asp	BEZ-235	4	123	120	0.216	0.262
p.Gly13Asp	Sirolimus	4	123	116	0.019	0.040
